# Atmospherically Stable Poly(Heptazine Imide) Composites

**DOI:** 10.1021/acsomega.6c00037

**Published:** 2026-03-04

**Authors:** Tatsushige Izumi, Ryoma Hayakawa, Momoka Isobe, Ryosuke Ohnuki, Yutaka Wakayama, Shinya Yoshioka, Kaname Kanai

**Affiliations:** † Department of Physics and Astronomy, Faculty of Science and Technology, 13258 Tokyo University of Science, 2641 Yamazaki, Noda, Chiba 278-8510, Japan; ‡ Research Center for Materials Nanoarchitectonics (MANA), National Institute for Materials Science (NIMS), 1-1 Namiki, Tsukuba, Ibaraki 305-0044, Japan

## Abstract

Metal poly­(heptazine imide) (MPHI), a two-dimensional carbon nitride polymer containing monovalent metal ions (M^+^), has recently attracted attention as a novel visible-light-driven photocatalyst. It exhibits photochromism, changing from yellow to blue-green upon light irradiation, regardless of the metal species, and is known to enhance ionic conductivity. Consequently, it has the potential to serve as a novel photoresponsive ionic conductor. However, the excited (color-changed) state that exhibits ionic conductivity is easily deactivated by atmospheric or dissolved oxygen in solution, making its application in actual devices challenging. Therefore, in this study, we developed a composite, protonated poly­(heptazine imide) (HPHI):poly­(vinyl alcohol) (PVA), by dispersing HPHI prepared by the acid treatment of potassium poly­(heptazine imide) into a matrix of the insulating polymer PVA, which possesses high oxygen-blocking properties. HPHI:PVA can maintain a color-changed state for extended periods, even in air, while sustaining a low electrical resistance state. The time constant derived from the decay curve of HPHI:PVA’s absorbance over time is six times longer than that reported for HPHI composites using poly­(methyl methacrylate) in previous studie. The duration of this color-changed state can be controlled by varying the degree of PVA saponification or temperature. Furthermore, a detailed investigation of the dependence of the electrical properties of HPHI:PVA on the percentage of HPHI revealed that proton conduction in HPHI:PVA arises from the percolation of poly­(heptazine imide) particles within the composite. This finding also provides fundamental information regarding the ion-conduction mechanism in other MPHI composites. This study serves as an important guideline for the future development of new MPHI composites and applied research.

## Graphical abstract

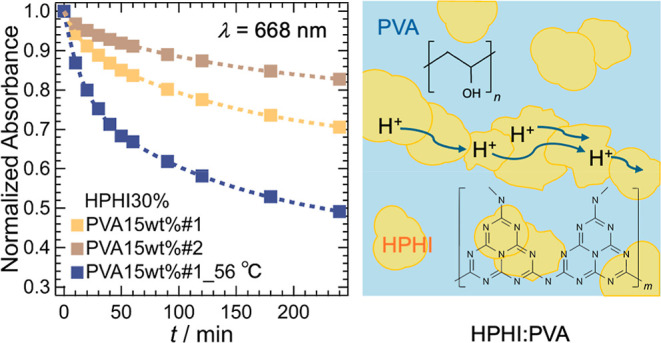

## 1 Introduction

Metal poly­(heptazine imide) (MPHI), a two-dimensional carbon nitride (CN) polymer containing monovalent metal ions (M^+^), has recently attracted attention because of its high photocatalytic activity compared with those of other CN polymers and its ability to exhibit dark photocatalytic activity by consuming the accumulated charge under visible-light irradiation.^2–6^ Figure 1a shows the molecular structure of potassium poly­(heptazine imide) (KPHI, M=K), while Figure 1b shows the corresponding protonated poly­(heptazine imide) (HPHI) obtained by protonating MPHI. Most MPHI materials exhibit photochromism under light irradiation, transitioning in color from yellow to blue-green regardless of the incorporated metal.^2,4–7^ Several studies have explored how this photochromic behavior contributes to the dark photocatalytic activity of MPHI.^1,7–9^ We previously reported that light irradiation induces the desorption of metal ions from MPHI, resulting in a sharp increase in the ionic conductivity. This ion desorption from the poly­(heptazine imide) (PHI) framework alters the electronic state of PHI, giving rise to photochromism.^1,9,10^ It is understood that when ions desorbed recombine onto the PHI framework, MPHI reverts to yellow. To date, MPHI has been studied not only as a photocatalyst but also for its potential in photochromism-based color switching and light-responsive ion conductivity. The estimated ion conductivity of KPHI under light irradiation, as measured using KPHI nanosheets, reaches a maximum of approximately 7.0 × 10^–8^ S/cm at room temperature.^1^ In contrast, the ion conductivity of PEO:Ga-LLZO, a representative solid polymer electrolyte, reaches a maximum of 7.2 × 10^–5^ S/cm at 30 °C.^11^ Thus, while the ionic conductivity of KPHI is extremely low compared to solid polymer electrolytes like PEO, it possesses the unique functionality of light-responsive ion conductivity. For example, MPHI has been used to develop anticounterfeiting films and oxygen colorimetric sensors for food applications.^12–14^ In addition, research on the photo-Seebeck effect of MPHI has also been reported.^15^ However, because the excited state of MPHI is easily deactivated by oxygen,^7,8,12,13^ challenges remain for its practical use as a light-responsive, ion-conductive, and photochromic material. Therefore, composites have been developed in which MPHI is dispersed in ionic liquids, poly­(methyl methacrylate) (PMMA) and poly­(vinyl acetate) (PVAc).^9,10,13,16^ These composites exhibit relatively stable MPHI-derived photochromism and light-responsive ion conductivity because the MPHI encapsulated within the matrix does not come into direct contact with atmospheric oxygen. However, even in these composites, the influence of ambient oxygen cannot be fully eliminated, and the photochromic coloration produced by light irradiation typically persists for only a few hours at most.^10,13,16^ Therefore, developing MPHI composites with enhanced resistance to oxygen is essential. Furthermore, the ion-conduction mechanisms operating in these composites remain insufficiently understood. Understanding the ion-conduction mechanism of MPHI composites is essential for developing new materials with superior ion conductivity.

**Figure 1:**
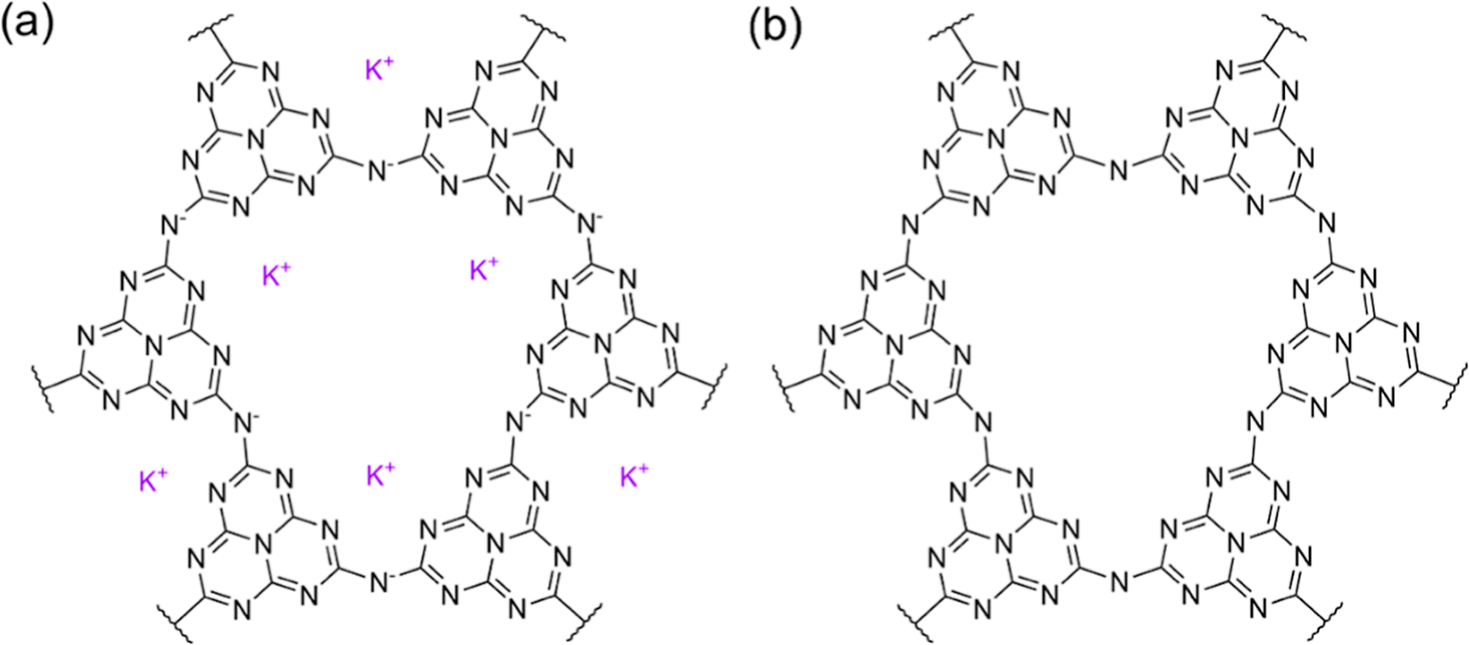
(a) Molecular structure of potassium poly­(heptazine imide) (KPHI). The diagram shows three K^+^ ions inside the pore; however, in reality, KPHI may contain an average of one K^+^ per unit.^1^ In that case, two nitrogen atoms are protonated on average. (b) Molecular structure of protonated poly­(heptazine imide) (HPHI).

In this study, we developed a novel MPHI composite by dispersing MPHI in a poly­(vinyl alcohol) (PVA) matrix. PVA has high chemical resistance and is water-soluble, making it easy to handle. In addition, it possesses excellent oxygen-blocking properties.^17–19^ Furthermore, PVA is an ion conductor and widely used in applications such as polarizing films, solid polymer electrolytes, pharmaceuticals, and adhesives.^20–23^ HPHI was used as the MPHI dispersed in PVA (hereinafter, the HPHI composite with PVA as the matrix is referred to as HPHI:PVA). HPHI was prepared by the ion exchange of K^+^ in KPHI with H^+^ through acid treatment.^16^ HPHI:PVA was found to maintain its color-changed state, which is indicative of the excited state of HPHI, for a longer period than other MPHI composites. Furthermore, it was revealed that the duration of the excited state of HPHI can be controlled by adjusting parameters such as the degree of saponification (SD), which governs the free volume in PVA, as well as the temperature. In addition, electrical property measurements were performed to investigate the electrical conductivity of HPHI:PVA. Because HPHI does not contain metal ions and PVA exhibits proton conductivity,^24–26^ HPHI:PVA is expected to exhibit proton conductivity. Electrical conductivity measurements conducted while varying the amount of dispersed HPHI within the composite revealed that, contrary to expectations, protons released from the PHI framework under light irradiation were conducted not through the PVA matrix but through the dispersed HPHI particles in the composite. In other words, it was found that proton conduction in HPHI:PVA exhibits so-called “percolation conduction.” The fundamental properties of HPHI:PVA clarified in this study provide important guidelines for future research on MPHI composites.

## 2 Experimental Section

### 2.1 Preparation of KPHI

Melon was prepared as the KPHI precursor. A quartz test tube and quartz tube for calcination were first heated at 700 °C for 45 min in a tube furnace (JTEKT THERMO SYSTEMS Co., Ltd., KTF035N1). Melamine (3.0 g, 99.0%, Wako Pure Chemicals Co., Ltd., 139-00945) was then placed in a quartz test tube, covered with aluminum foil containing a single pinhole at the center (≈0.6 mm in diameter), and secured with a tungsten wire. The test tube was inserted into the quartz tube. Synthesis was performed under a nitrogen atmosphere (purity: 99.99995%) using the following temperature program: heating at 1 °C min^–1^ to 550 °C, holding for 5 h, and subsequently cooling at 2 °C min^–1^ to room temperature.

To synthesize KPHI, a quartz test tube and quartz tube were first heated at 700 °C for 45 min. Then, the synthesized melon (0.3 g) and KSCN (0.15 g, purity: 98.0%, FUJIFILM Wako Pure Chemicals Co., Ltd.; 164-04555) were mixed and placed in a calcination boat. The mixture was covered with aluminum foil, placed in a quartz test tube, and secured with a tungsten wire. Synthesis was performed under a nitrogen atmosphere (purity: 99.99995%) using the following temperature program: heating at 30 °C min^–1^ to 400 °C and holding for 1 h, followed by heating at 30 °C min^–1^ to 500 °C and holding for 30 min, and then cooling at 2 °C min^–1^ to room temperature. The product was washed four times with pure water (FUJIFILM Wako Pure Chemical Corporation, 161-08247) and separated by centrifugation. Finally, the samples were dried in a desiccator to obtain KPHI as a yellow powder.

### 2.2 Preparation of HPHI

Sulfuric acid (2 mL, sulfuric acid content: 97%; FUJIFILM Wako Pure Chemical Corporation, 190-04675) was added to a graduated cylinder containing pure water (35 mL). Additional pure water was added to bring the total volume to 40 mL, yielding a dilute sulfuric acid solution. This solution was transferred to a beaker, and KPHI (300 mg) was added. The mixture was stirred at 500 rpm for 30 min, followed by ultrasonic treatment for 10 min. The mixture was then transferred to a centrifuge tube and centrifuged for 2 min. The supernatant in the centrifuge tube was discarded, pure water was added, and the mixture was centrifuged again for 2 min; this procedure was repeated twice. After discarding the supernatant, pure water was added, and the mixture was centrifuged for 10 min. Finally, the sample was dried in a desiccator to obtain HPHI as a white powder.

### 2.3 Preparation of HPHI:PVA

PVA (average polymerization degree ≈900–1100; partially saponified, saponification degree 86%–90%; fully saponified, saponification degree ≥96%; FUJIFILM Wako Pure Chemical Corporation, 9002-89-5) was added to pure water and stirred at 85–100 °C while heating until the PVA completely dissolved, at approximately 300 rpm. HPHI was then added, and the mixture was heated at 85 °C while stirring at 300 rpm for several hours. For electrical measurements, the samples were prepared by dispensing the above aqueous solution (40 μL) between Au electrodes spaced 2 mm apart, followed by heating and drying in the dark at 50 °C for 3 days. The Au electrodes were fabricated by sputter deposition; first, 5 nm of Cr was deposited onto a glass substrate, followed by 50 nm of Au.

### 2.4 Characterization

An LED (HAYASHI-REPIC, LA-HDF 100NA) was used as the visible light source for the PHI photochromism. The light spectrum of this source is shown in Figure S1 in Supporting Information. Ultraviolet–visible (UV–vis) absorption spectroscopy was performed using a spectrometer (JASCO Corporation, V-670 equipped with an integrating sphere). The film samples were prepared on quartz glass plates, and the reflectance of the light was measured. A film heater (HET-ON4; KYOHRITSU ELECTRONIC INDUSTRY Co., Ltd.) was used to heat the samples. Electrical measurements were conducted using a source measurement unit B2912B (Keysight Technology). A visible light source (LED head unit CL-H1-405-9-1-B with controller CL-1503; ASAHI SPECTRA Co., Ltd.) was used for light irradiation during the electrical measurements. The relative humidity, recorded using a precision thermo-hygrometer (HD-120, Crecer Co., Ltd.), ranged from 40%RH to 47%RH during the measurements. Electrochemical impedance spectroscopy (EIS) was conducted using an LCR meter (Keysight Technologies, E4980A). All measurements were performed using a four-probe system at room temperature under atmospheric conditions. The pyZwx software was employed to obtain the impedance spectrum and fit it using appropriate equivalent circuit models.^27^ These electrical measurements were performed on HPHI:PVA films formed on Au electrodes. The Au electrode had a spacing of 2 mm and a thickness of 50 nm.

## 3 Results and Discussion

Figure 2a shows the structure of PVA. In general, PVA is synthesized from its precursor, PVAc, through a process called saponification. During this process, the acetic acid groups in the side chains of PVAc are hydrolyzed to form hydroxyl groups in the side chains of PVA. The extent of this hydrolysis is referred to as the SD. When the degree of polymerization of PVAc is denoted as *m* and that of PVA as *n*, the SD [%] is defined by the following equation

1 $$SD=\frac{n}{n+m}\times 100$$

in this paper, the copolymer of PVA and PVAc shown in Figure 2a is referred to simply as “PVA.” In addition, PVA with an SD in the range of 86%–90% is referred to as “partially saponified PVA” (PVA#1), and PVA with an SD exceeding or equal to 96% is referred to as “fully saponified PVA” (PVA#2). As the SD increases, the steric hindrance from the acetate group of PVAc decreases, facilitating the aggregation of PVA molecules and formation of hydrogen bonds. As a result, in fully saponified PVA, the glass transition temperature increases and the segment motion of the polymer chains is suppressed. Consequently, oxygen intrusion and diffusion into PVA can be effectively prevented. Therefore, the SD is an important parameter that determines the oxygen permeability of PVA.^28^

**Figure 2:**
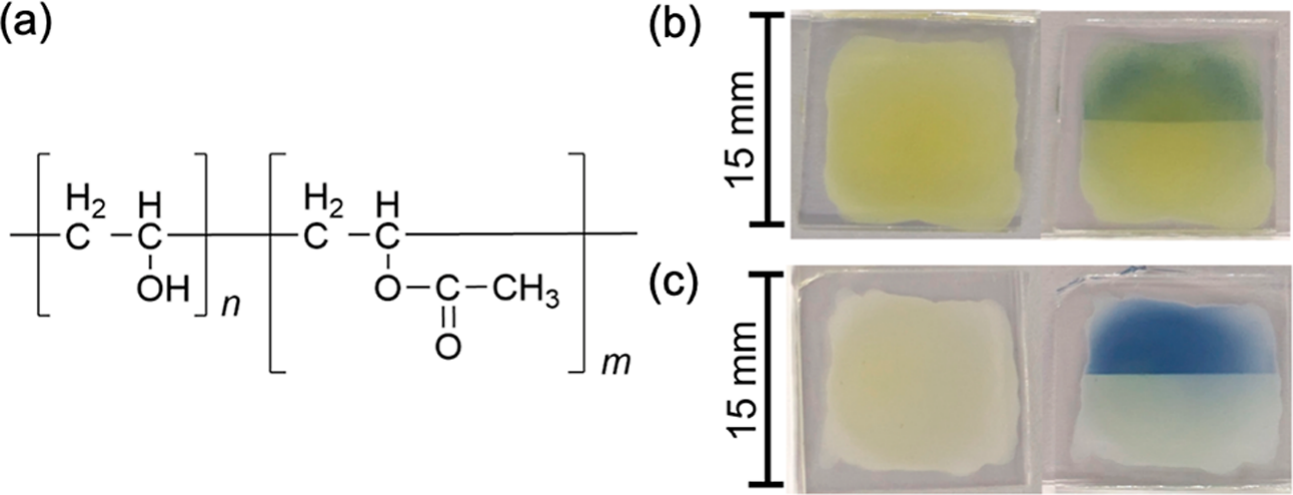
(a) Molecular structure of the copolymer of poly­(vinyl alcohol) (PVA) and polyvinyl acetate (PVAc). The degree of polymerization of the PVAc unit is denoted by *m*, and that of the PVA unit by *n*. (b) Left: KPHI:PVA#1 before light irradiation; right: KPHI:PVA#1 after light irradiation. Light was irradiated on the upper half of the sample. PVA#1 refers to partially saponified PVA with an SD in the range of 86%–90%. (c) Left: HPHI:PVA#1 before light irradiation; right: HPHI:PVA#1 after light irradiation. Light was irradiated on the upper half of the sample.

Figure 2b,c show photographs of the KPHI:PVA#1 and HPHI:PVA#1 films, respectively. These films were prepared by dispensing a KPHI:PVA#1 or HPHI:PVA#1 solution (50 μL) onto a glass substrate, spreading it evenly, and drying it in the dark at 50 °C for 3 days. As shown in the photographs, when light is irradiated on the upper half of each sample, photochromism causes KPHI:PVA#1 to turn blue-green and HPHI:PVA#1 to turn dark blue (hereinafter, the state in which the color has changed due to photochromism is referred to as the “color-changed state”). As shown in Figure S2 in Supporting Information, KPHI:PVA#2 and HPHI:PVA#2 also exhibited color changes in response to light. The difference in color between the KPHI and HPHI composites after light irradiation is attributed to differences in their electronic states. Previous studies have shown that HPHI has an energy gap approximately 0.3 eV larger than that of KPHI.^16^ The color-changed states of these composites gradually revert to their original yellow states over several days. This indicates that composites using PVA as a matrix maintain their color-changed states for a longer period than other PHI composites. In addition, PVA is water-soluble, facilitating the formation of flexible composite films, and exhibits excellent adhesion to substrates such as glass, thereby significantly improving handling properties.

Figure 3 presents the time-dependent UV–vis spectra of HPHI:PVA#1 and HPHI:PVA#2. Figure 3a shows the UV–vis spectra of HPHI:PVA#1. Immediately after 5 min of light irradiation, an absorption band with a peak wavelength of approximately 668 nm appeared owing to the photochromism of HPHI. Over time, the intensity of this absorption band gradually decreased; however, as shown in the figure, it retained substantial intensity even after 240 min. This duration is significantly longer than that reported for composites such as KPHI:PMMA or KPHI:PVAc in previous studies.^13,16^ In general, the free volume influences the gas permeability of polymeric materials. This quantity is determined by factors such as the cohesive forces within the polymer and the extent of steric hindrance arising from its side chains. For example, it is known that bulky side chains on polymers inhibit the dense packing of polymer chains, thereby increasing the free volume.^29^ In general, a larger free volume allows gas molecules to permeate and diffuse more easily through the polymer film. Therefore, when the steric hindrance of the side chains is large, gas permeability tends to increase.^29^ PVA exhibits a low free volume owing to its high crystallinity, which results from strong aggregation driven by hydrogen bonding and its short side chains. Furthermore, a higher SD reduces the presence of side chains with significant steric hindrance originating from the acetate groups derived from PVAc. This promotes stronger aggregation of the PVA molecules, enhances the crystallinity, and further decreases the free volume. As a result, the oxygen permeability decreases.^28,30,31^ Figure 3b shows the UV–vis measurement results for the HPHI:PVA#2 composite, which was prepared using fully saponified PVA (PVA#2). Compared with HPHI:PVA#1 shown in Figure 3a, the rate of decrease in the intensity of the 668 nm absorption band was slower. This result suggests that using PVA with a high SD and low free volume enhances oxygen blocking, thereby allowing longer-lasting color-change retention.

**Figure 3:**
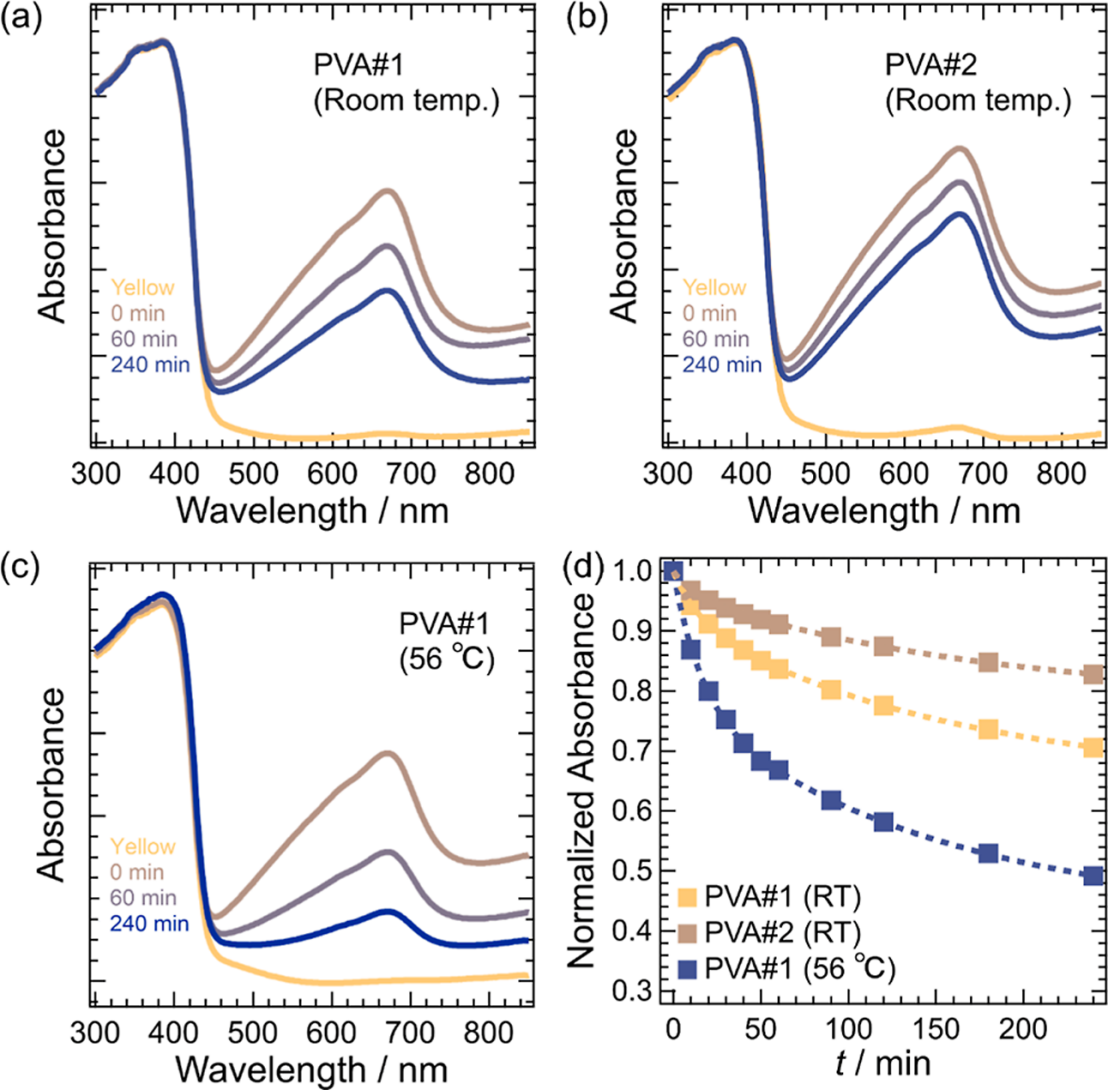
(a) Time-dependent ultraviolet–visible (UV–vis) spectra of a sample containing 30% HPHI relative to the mass of PVA#1 (PVA#1). The sample was prepared from a 15 wt % aqueous solution of PVA#1. “Yellow” denotes the spectrum before light irradiation; “0 min” denotes the spectrum recorded immediately after 5 min of light irradiation; “60 min” and “240 min” denote the spectra measured 60 and 240 min, respectively, after irradiation was stopped. All measurements were performed at room temperature. (b) Time-dependent UV–vis spectra of a sample containing 30% HPHI relative to the mass of PVA#2 (PVA#2). The sample was prepared from a 15 wt % aqueous solution of PVA#2. The legend is the same as in panel (a). Measurements were performed at room temperature. (c) Time-dependent UV–vis spectra of PVA#1. The legend is the same as in panel (a). Measurements were performed at 56 °C. (d) Time-dependent changes in the absorbance at *λ* = 668 nm in the UV–vis spectra shown in panels (a–c) after light irradiation. Absorbance values are normalized to the absorbance immediately after irradiation (0 min).

Heating polymers can increase the thermal motion of the polymer chains, potentially increasing their free volume. Therefore, as the temperature rises, the free volume of the polymer expands, leading to a higher oxygen permeability. Figure 3c shows the time-dependent UV–vis spectrum of HPHI:PVA#1 measured at 56 °C. Compared with the results in Figure 3a, the decrease in the intensity of the 668 nm absorption band in Figure 3c occurs more rapidly. This result indicates that the color-changed state of HPHI:PVA#1 disappeared more rapidly because the free volume of PVA increased at high temperatures, facilitating oxygen permeation.^31–33^ In fact, the glass transition temperature of PVA#1 has been reported to be around 53 °C.^28^

In general, moisture-absorbing polymer films such as PVA exhibit a rapid increase in free volume under high humidity.^34–37^ As water molecules are absorbed into the polymer film, they break the hydrogen bonds between PVA molecules while increasing hydrogen bonding between PVA and water molecules, as well as between the water molecules themselves. Consequently, the intermolecular bonds between the PVA molecules weaken, causing the PVA chains to separate. This separation allows the chains to move more freely, thereby increasing the free volume. Figure S3 in Supporting Information shows the time-dependent color change of HPHI:PVA at 60 °C after light irradiation. Samples heated under high-humidity conditions exhibited more rapid color recovery than those heated under ambient conditions. This clearly demonstrates that the retention of the color-changed state in HPHI:PVA strongly depends on the oxygen permeability of PVA. Figure 3d shows the time dependence of the absorbance at *λ* = 668 nm in the UV–vis spectra of Figure 3a–c after light irradiation. The time constants *τ* obtained from fitting these decay curves using eq 2 are summarized in Table 1.

2 $$A=A_{0}+A_{1}e^{\left(-t/\tau_{1}\right)}+A_{2}e^{\left(-t/\tau_{2}\right)}$$

where *A*_0_ corresponds to the absorbance of HPHI:PVA in the yellow state prior to light irradiation.

**Table 1:** Parameter Values Obtained by Fitting the Absorbance at *λ* = 668 nm in Figure 3d as a Function of Time after Light Irradiation Using eq 2

	*A*_0_	*A*_1_	*τ*_1_ (min)	*A*_2_	*τ*_2_ (min)
PVA#1 (RT)	0.288 ± 0.039	(9.8 ± 3.4) × 10^–2^	29 ± 4	0.605 ± 0.006	(8.1 ± 0.5) × 10^2^
PVA#2 (RT)	0.294 ± 0.033	(4.5 ± 0.6) × 10^–2^	27 ± 5	0.659 ± 0.004	(16.5 ± 0.8) × 10^2^
PVA#1 (56 °C)	0.260 ± 0.008	0.278 ± 0.020	26.2 ± 3.1	0.464 ± 0.020	(3.40 ± 0.36) × 10^2^

As shown in Table 1, the time constant *τ*_1_ of the transient component shows little difference between HPHI:PVA#1 and HPHI:PVA#2. In contrast, the time constant *τ*_2_ of the delayed component for HPHI:PVA#2 is more than twice that of HPHI:PVA#1. The *τ*_2_ of PVA#2 is approximately six times that of the *τ*_2_ of HPHI:PMMA (*τ*_2_ = (2.75 ± 0.35) × 10^2^ min) reported in the previous work.^16^ Furthermore, the *τ*_2_ of PVA#1 heated to 56 °C is less than half that of HPHI:PVA#1 measured at room temperature.

Figure 4 shows the effects of light irradiation on the proton conductivities of HPHI:PVA#1 and HPHI:PVA#2 (*IT* characteristics, Figure 4a), as well as their current–voltage characteristics (Figure 4b). During the *IT* measurements, a voltage of 5 V was applied to the samples.

**Figure 4:**
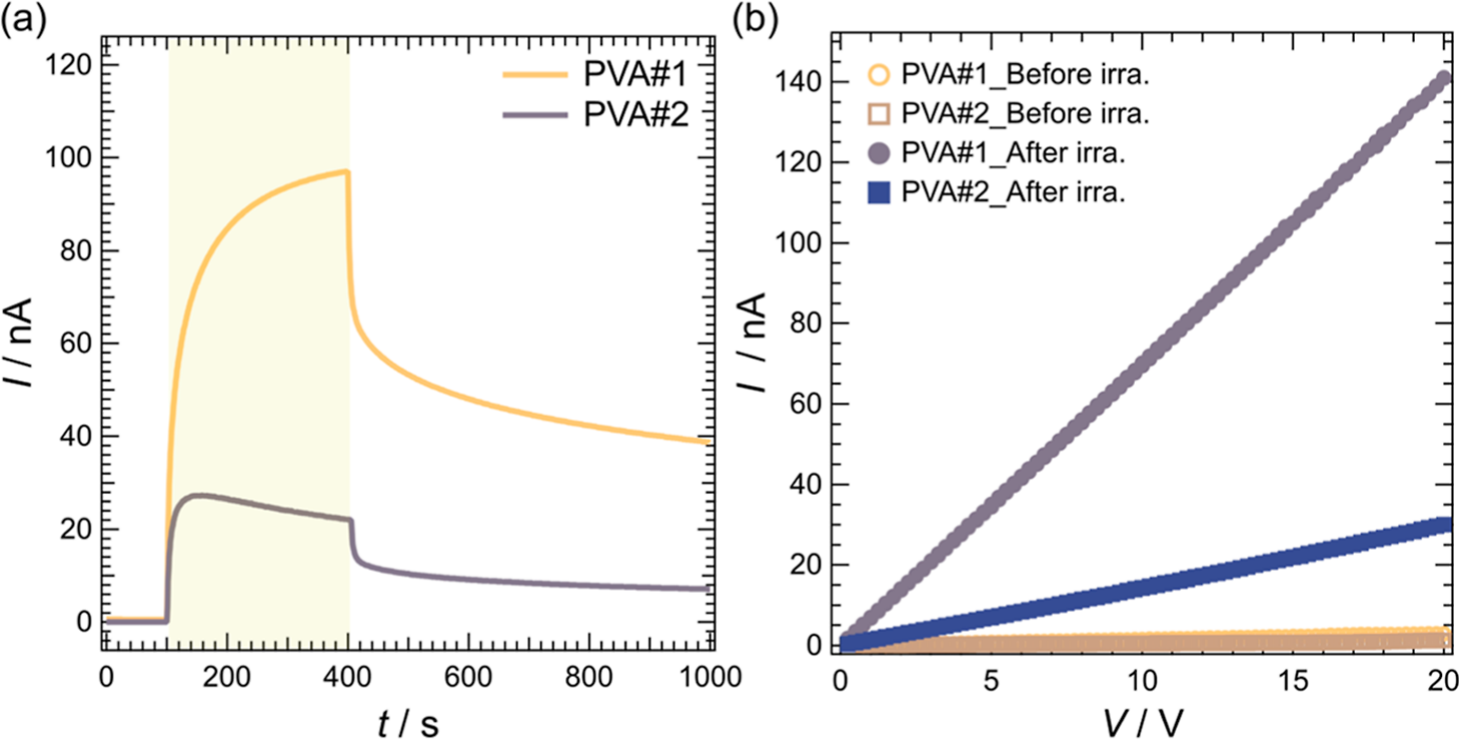
(a) Electric current as a function of time for HPHI:PVA#1 (PVA#1) and HPHI:PVA#2 (PVA#2). The samples measured here were prepared using a 6 wt % aqueous solution of PVA. Measurements were performed by applying 5 V to the samples. The light yellow area in the graph indicates the period during which light was irradiated onto the yellow sample surface. (b) Electric current as a function of applied voltage for HPHI:PVA#1 (PVA#1_Before irra. and PVA#1_After irra.) and HPHI:PVA#2 (PVA#2_Before irra. and PVA#2_After irra.) before and after 5 min of light irradiation.

From the *IT* characteristics shown in Figure 4a, both HPHI:PVA#1 and HPHI:PVA#2 exhibited negligible currents before light irradiation. Since the HPHI:PVA composite consists of HPHI dispersed within the insulating polymer PVA, electrons or holes cannot be injected as carriers from the electrodes, and electrical conduction occurs via proton conduction. Immediately after light irradiation, the current due to proton conduction increased sharply in both samples but did not saturate. During light irradiation, the photocurrent gradually increased for HPHI:PVA#1 but gradually decreased for HPHI:PVA#2. HPHI:PVA#1 exhibited an approximately 4-fold greater current increase under light irradiation compared with that of HPHI:PVA#2. This difference is attributed to the variation in the amount of moisture absorbed by the PVA. Yamada et al. reported that, within the SD range of 99% to 77%, PVA with a higher SD exhibits a lower rate of moisture absorption.^38^ This is because PVA#2 has higher crystallinity than PVA#1, resulting in less free volume for water molecules to penetrate. Consequently, PVA#1 contains more moisture than PVA#2. As discussed in detail below, the water molecules within the composite play a crucial role in proton conduction. This likely explains why PVA#1 exhibits a higher photocurrent owing to its greater proton conductivity, as shown in Figure 4a.

For both samples, after light irradiation was stopped, the current did not decrease immediately, exhibiting persistent photoconductivity (PPC). PPC has also been observed in composites such as KPHI with ionic liquids or KPHI with PMMA.^10,16^ In general, proton conduction occurs primarily via the Grotthuss mechanism, which involves a series of proton jumps along a hydrogen-bond network, and the vehicle mechanism, where the oxonium ion itself, attached to a water molecule, moves through the medium. Based solely on the electrical properties shown in Figure 4a, it is impossible to determine which mechanism governs proton conduction in HPHI:PVA; however, the observation of PPC provides an important clue. To understand the origins of PPC, two points must be considered. First, when the light irradiation of HPHI:PVA was stopped, the color of HPHI:PVA gradually changed from blue back to yellow, and the current decreased in synchrony with this change.^1,10^ This indicates that protons released from the PHI framework by light irradiation do not recombine with the PHI framework immediately after the irradiation is stopped, but rather do so gradually. Second, if the proton conduction mechanism in HPHI:PVA were dominated by the vehicle mechanism, it would suggest that the mobility of oxonium ions (H^+^/H_3_O^+^) within the composite is likely very low. For example, partial proton detachment, which ionizes the PHI framework upon light irradiation, may distort the PHI structure and inhibit H^+^/H_3_O^+^ movement. Therefore, H^+^/H_3_O^+^ moving within HPHI must experience significant scattering and trapping by the negatively charged PHI framework. Combining the two points above, the following scenario based on the vehicle mechanism can be proposed: First, light irradiation causes a sharp increase in the number of protons within HPHI:PVA, leading to a rapid rise in conductivity. During this process, protons are conducted within the composite in the form of oxonium ions. This hypothesis is plausible because in MPHI materials, including KPHI, ion conduction originates from the hydration of metal ions,^39^ which explains why PVA#1 with a high water content exhibits higher photoconductivity. However, the mobility of the oxonium ions is extremely low because they are scattered and trapped by the charged and distorted PHI framework. Upon cessation of light irradiation, protons slowly recombined with the PHI framework, causing the proton number to gradually decrease and thereby inducing PPC. Because it is unlikely that the oxonium ion releases a single proton directly, the proton may instead be transferred between water molecules and ultimately transferred to the PHI framework, leading to its reincorporation into the PHI framework.

Figure 4b shows the changes in the current–voltage characteristics of HPHI:PVA#1 and HPHI:PVA#2 before and after light irradiation. For both samples, only a very small current flowed before light irradiation; however, the current increased significantly after light irradiation. The current observed after light irradiation increases linearly over a wide range of applied voltages. This indicates that only proton conduction occurred in the composites without the injection of electrons or holes from the electrodes into the HPHI:PVA. If carriers were injected into the composites from the electrodes, the current would not increase linearly from approximately 0 V. Instead, a threshold voltage that produces distinct current increases should be observed.^1^

Assuming that protons are conducted through HPHI:PVA via a vehicle mechanism, the next question is how the conduction path is formed. To address this, Figure 5a shows the measured electrical current for samples with different mass fractions of HPHI in HPHI:PVA. The *Δ* *I* in Figure 5a represents the difference between the maximum current observed during 5 min of light irradiation of HPHI:PVA#1 and the current before irradiation. In Figure 5a, the *Δ* *I* is plotted against the mass ratio *x* of HPHI relative to the total sample mass.

**Figure 5:**
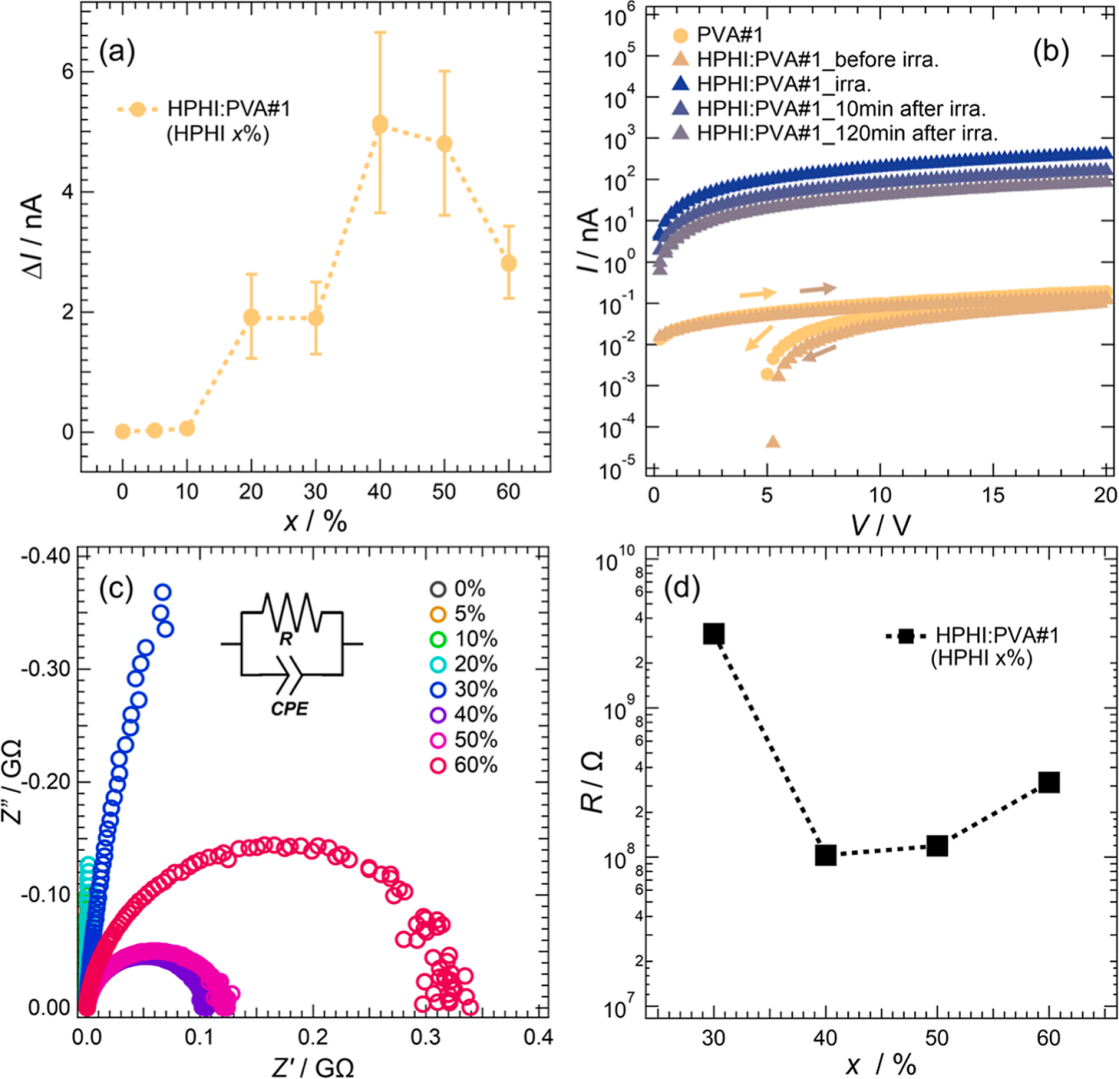
(a) Increase in current (*Δ* *I*) before and after light irradiation plotted against the HPHI percentage (*x*) in HPHI:PVA#1. The *IT* measurement was performed by applying 1 V to the sample. Measurements were taken three times within a relative humidity range of 40%RH to 47%RH, and the average values of *Δ* *I* are plotted. (b) *IV* measurement results for HPHI:PVA#1 (*x* = 50%). For reference, the *IV* measurement result for PVA#1 (*x* = 0%) is also shown. Measurements for HPHI:PVA#1 were taken before light irradiation, under light irradiation, 10 min after light irradiation, and 2 h after light irradiation. (c) Electrochemical impedance spectroscopy results for HPHI:PVA#1 samples. Nyquist plots measured as a function of *x*. The horizontal axis *Z*′ shows the real part of the impedance, and the vertical axis *Z*′′ shows the imaginary part of the impedance. (d) Ion resistances *R* of the samples calculated from the Nyquist plots plotted against *x* (logarithmic axis).

In the region where the HPHI ratio is low (*x* ≤ 10%), the *Δ* *I* is very small. The *Δ* *I* gradually increases from approximately *x* = 20% HPHI, reaches high values at *x* = 40% and 50%, and then decreases when *x* is increased to 60%. These results suggest the following possibilities regarding the proton conduction mechanism in the composite. When *x* is low, a slight increase in the *Δ* *I* is observed upon light irradiation. This indicates that HPHI functions as a proton source for the composite.^40^ The *IV* characteristics of PVA#1 shown in Figure 5b indicate that the current for PVA alone is very small, on the order of 10^–1^ to 10^–2^ nA. This means that proton conduction due to protons supplied by water or other components contained in PVA is essentially absent. Figure 5a shows that photoconduction occurs when *x* reaches 20%. Proton conduction arises when protons are released from the PHI framework upon irradiation with light. The results shown in Figure 5a provide insight into the formation of proton conduction paths within the composite. A key point is that, within a small *x* range, photoconduction does not increase proportionally with *x*. If the protons supplied by HPHI upon light irradiation were conducted directly through PVA, the *Δ* *I* would be expected to increase proportionally with *x*. The fact that the *Δ* *I* increases when *x* exceeds a threshold value of 20% indicates that protons supplied by HPHI cannot be conducted below this value. In other words, when the percentage of HPHI in the composite exceeds 20%, it can be considered that percolation conduction occurs because the HPHI domains become interconnected within the composite. Protons generated within the HPHI under light irradiation are thought to hydrate into oxonium ions and migrate through channels in the PHI framework without conducting through PVA. Therefore, for the composite to exhibit photoconductivity, the HPHI particles must form connected pathways that bridge the gap between the negative and positive electrodes.

The remaining question is why, after the *Δ* *I* reaches its maximum at 40%, further increasing *x* to 60% leads to a decrease in the *Δ* *I*. As the percentage of HPHI in the composite increases, HPHI particles near the complex surface can no longer be fully encapsulated by PVA and become readily exposed to oxygen. Furthermore, these surface HPHI domains absorb light, reducing the amount that reaches the bulk of the composite. HPHI in the surface region, even when absorbing light and undergoing photochromism, is rapidly de-excited by oxygen, thereby suppressing efficient proton generation. Therefore, as *x* increases, although the connectivity of the HPHI particles establishes sufficient proton-conduction pathways within the composite, but the actual number of protons that pass through these pathways might decrease. However, since this is currently only a hypothesis, we need to devise a method to experimentally verify it in the future.

Experimental results supporting the hypothesis that proton conduction in HPHI:PVA occurs through percolation were also obtained from the EIS measurements. Figure 5c presents the EIS data for HPHI:PVA#1 with varying HPHI percentages. The left and bottom axes correspond to the imaginary and real components of the impedance, respectively, and the measurement frequency ranged from 20 Hz to 2 MHz. The legend in the figure indicates the percentages of HPHI added to the HPHI:PVA mixtures. Results for HPHI contents ranging from 0% to 20% are very similar; the impedance is almost entirely dominated by the imaginary component, resulting in a Nyquist plot that does not form a circle. This behavior corresponds to the capacitive response of the insulating PVA film, as HPHI does not form connected pathways for proton conduction within the composite. Next, increasing the HPHI content to 30%–60% significantly reduces the imaginary component of the impedance, leaving almost exclusively the real component, and the HPHI exhibits a response similar to that of a resistor. Generally, when no external voltage is applied, most organic materials exhibit capacitor-like behavior because they lack charge carriers capable of responding to the applied AC voltage. However, when carriers capable of responding to external electric fields, such as mobile ions, are present within a material, it exhibits resistor-like behavior. Similar to many other materials, HPHI:PVA exhibited a capacitor-like response before light irradiation. However, upon light irradiation, protons are released from the heptazine skeleton and become mobile, causing the material to respond in a resistor-like manner. Figure 5c shows an equivalent circuit consisting of the ion resistance (*R*) of HPHI and a constant phase element. In the equivalent circuit, the diameters of the semicircles in the Nyquist plots represent the *R* values of the samples. The diameter of the semicircle changes dramatically with the HPHI percentage, indicating that *R* exhibits a strong dependence on the HPHI percentage. Figure 5d plots *R* as a function of *x*. These results show that HPHI:PVA#1 exhibits proton conduction at *x* ≥ 30%, reaches its lowest resistance at *x* = 40%, and then shows increasing resistance as *x* further increases. The dependence of *R* on *x* is consistent with that of the Δ*I* in Figure 5a. Therefore, when *x* is 30% or higher, these results support a percolation conduction mechanism, wherein HPHI particles within the composite bond together to form proton-conduction pathways. Furthermore, when *x* exceeds 50%, the scenario in which the number of protons actually conducted through these pathways decreases as the fraction of HPHI particles exposed on the composite surface increases is supported by the observation that *R* decreases as *x* increases beyond 50%.

## 4 Conclusion

In this study, we successfully developed a PHI:PVA composite using PVA, an insulating polymer, as the matrix. First, we demonstrated that HPHI:PVA exhibited a significantly improved retention time for the light-irradiation-induced color-changed state of HPHI compared with that of HPHI alone or previously reported HPHI:polymer composites. Next, we examined the factors contributing to the long-lasting color-changed state of PHI:PVA by analyzing changes in the retention time as a function of the SD of PVA and temperature. UV–vis measurements of PHI:PVA revealed that increasing the SD of PVA prolongs the lifetime of the color-changed state, whereas increasing the temperature shortens it. These results indicate that changes in the free volume, one of the parameters governing the oxygen permeability of PVA films, significantly affect the retention time of the color-changed state.

The measured electrical properties of HPHI:PVA led to important conclusions regarding the conduction pathways of protons within the composite. The irradiation-induced increase in the proton conductivity of HPHI:PVA was investigated as a function of the HPHI percentage in the composite. These results clearly demonstrate that protons released from HPHI are conducted within the HPHI phase rather than through PVA. This behavior corresponds to a percolation conduction mechanism, and the EIS measurement results support this conclusion.

The PHI:PVA developed in this study contributes to the development of novel MPHI composites. Furthermore, insights into the mechanism of proton conduction within the composite are important for materials design. In particular, the long-lived color-changed state realized in HPHI:PVA exhibited PPC in proton conduction, suggesting potential for the future development of novel devices that utilize its photoresponsive ionic conductivity.

## Supplementary Material



## Notes

The data includes the spectrum of the LED used for irradiating the samples, photographs of KPHI:PVA#2 and HPHI:PVA#2 before and after light irradiation, and photographs of HPHI:PVA#1 and HPHI:PVA#2 at 60 °C under different humidity conditions after light irradiation (PDF)
