# Supplementary material for: Atmospherically Stable Poly(Heptazine Imide) Composites
Source: ACS Omega. 2026 Mar 4;11(10):16835–43. doi: 10.1021/acsomega.6c00037 (PMC13000576; doi:10.1021/acsomega.6c00037)
Supplement: Supplementary file 1 [file ao6c00037_si_001.pdf]

Supporting Information

# Atmospherically stable poly(heptazine imide) composites

Tatsushige Izumi[1], Ryoma Hayakawa[2], Momoka Isobe[1], Ryosuke Ohnuki[1], Yutaka Wakayama[2], Shinya Yoshioka[1], Kaname Kanai[1,*]

[1]*Department of Physics and Astronomy, Faculty of Science and Technology, Tokyo University of Science, 2641 Yamazaki, Noda, Chiba 278-8510, Japan*

[2]*Research Center for Materials Nano architectonics (MANA), National Institute for Materials Science (NIMS), 1-1 Namiki, Tsukuba, Ibaraki 305-0044, Japan.*

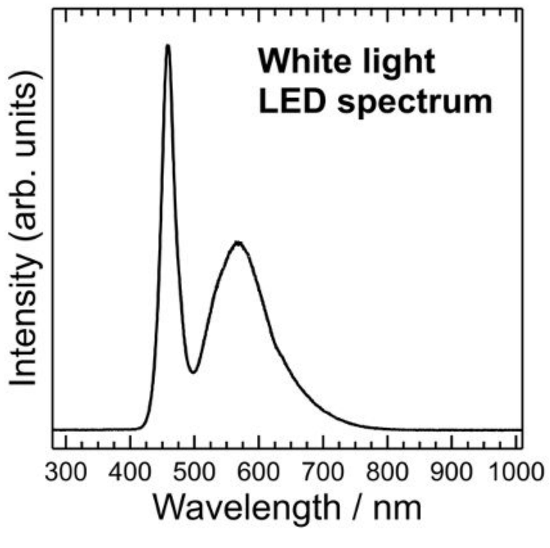


**Figure S1.** Spectrum of the LED used for irradiating the sample.

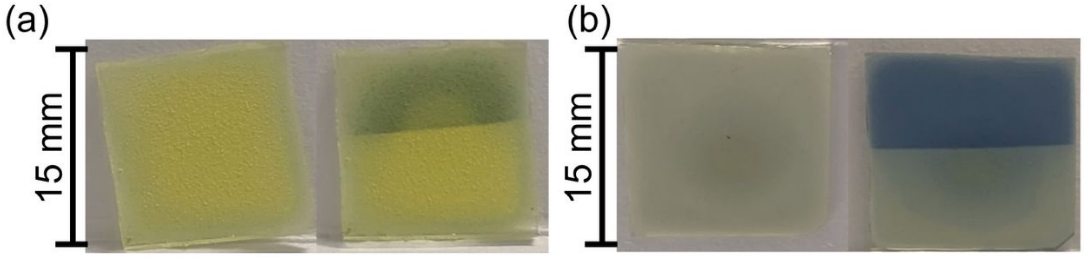


**Figure S2.** (a) The left photo shows KPHI:PVA#2 before light irradiation, and the right photo shows KPHI:PVA#2 after the light irradiation. Light was irradiated on the upper half of the sample. (b) The left photo shows HPHI:PVA#2 before light irradiation, and the right photo shows HPHI:PVA#2 after the light irradiation.

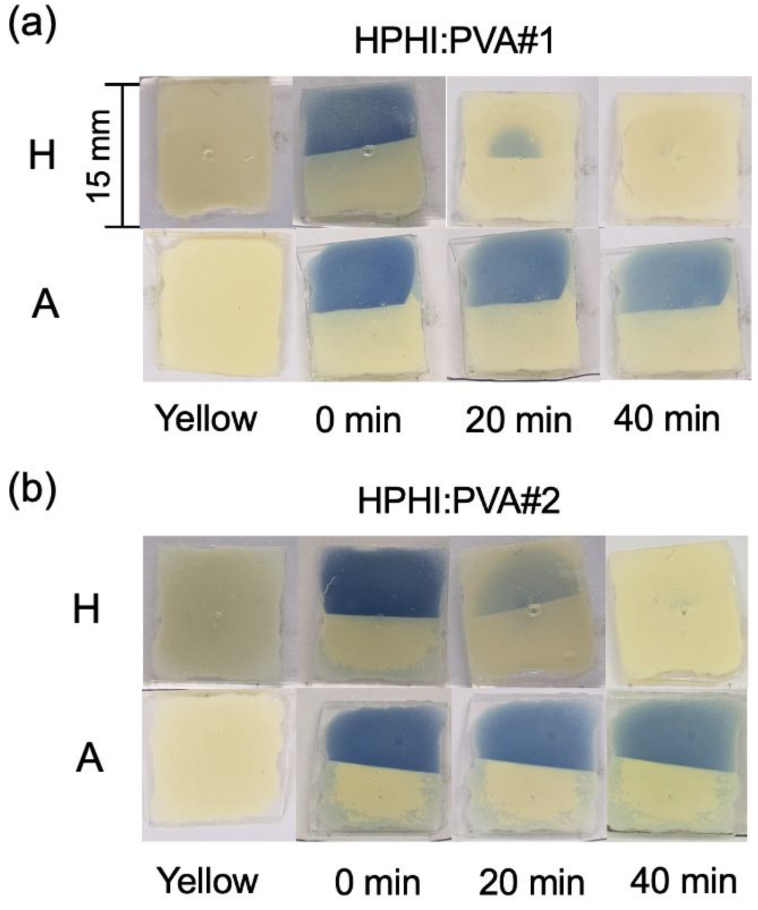


**Figure 3.** Photographs of HPHI:PVA#1 (a) and HPHI:PVA#2 (b) at 60°C after light irradiation. (a) shows the result for HPHI:PVA#1, and (b) shows the result for HPHI:PVA#2. "Yellow" indicates the photograph of the sample before light irradiation. Photographs taken immediately after light irradiation (0 min), 20 minutes later, and 40 minutes later are also shown. For both HPHI:PVA#1 and HPHI:PVA#2, samples heated under high humidity (H) exhibit faster decolorization compared to samples heated under ambient conditions (A).
